# Influence of Defense Mechanisms on Sport Burnout: A Multiple Mediation Analysis Effects of Resilience, Stress and Recovery

**DOI:** 10.3390/sports12100274

**Published:** 2024-10-11

**Authors:** Guillaume Levillain, Philippe Vacher, Yves de Roten, Michel Nicolas

**Affiliations:** 1 Laboratory of Psy-Drepi (EA 7428), University of Burgundy-Franche-Comté, Maison de l’université, Esplanade Erasme, BP 27877, CEDEX, 21078 Dijon, France; michel.nicolas@u-bourgogne.fr; 2 Laboratory CREAD, University of Brest, Place du Recteur Henri Le Moal, CS 24307, 35043 Brest, France; philippe.vacher@univ-brest.fr; 3 Institute of Psychotherapy, Department of Psychiatry, University Hospital Center, University of Lausanne, Route de Cery 1, 1008 Prilly, Switzerland; yves.deroten@chuv.ch

**Keywords:** defense mechanisms, sport burnout, resilience, stress, recovery, mediation analysis

## Abstract

The aims of this study were: (a) to explore the relationships between adaptive defense mechanisms (ADMs), maladaptive defense mechanisms (MADMs), stress, recovery, resilience, and sport burnout; and (b) to examine resilience, stress, and recovery as mediators of the relationship between defense mechanisms and burnout. One hundred and seventy-five athletes (*M* = 20.30 years, *SD* = 3.75) completed self-report questionnaires assessing defense mechanisms, resilience, stress, and recovery. Correlation analysis revealed that MADMs were positively associated with burnout, while ADMs had no significant link with burnout. Concerning mediation analysis, results showed a mediating effect of resilience in the relationship between ADMs and burnout. The findings also demonstrated a mediating effect of resilience and recovery in the relationship between MADMs and burnout. Our study highlighted that certain defenses categorized as adaptive might not be suitable in specific situations, thus underscoring the influence of mediating variables. The findings of mediation analysis demonstrated that resilience appears to serve as a particularly protective factor against burnout. On the contrary, MADMs would have a deleterious influence in the management of stress, which could lead to burnout. Coaches may consider fostering athletes’ resilience in conjunction with ADMs and implementing targeted psychological exercises to reduce the use of MADMs.

## 1. Introduction

Each domain in life requires intensive work and abnegation to perform and reach goals. In the sport context, athletes of all levels and from every sport are confronted with the pressure inherent to the competition [1,2]. The pursuit of victory and performance enhancement compels athletes to continually push their physical and mental limits [3]. This demanding environment, characterized by intense competition and the constant drive for improvement, places athletes at a heightened risk of experiencing chronic stress, a factor strongly associated with burnout [4]. The consequences of burnout for athletes could not only impact their overall performance but also their health and well-being [5]. Despite the acknowledged importance of mental health in the athletic realm, there remains a notable gap in the scientific literature concerning the exploration of defense mechanisms and their potential influence on burnout within the sports context.

Understanding how defense mechanisms contribute to or protect against burnout in the unique context of sports could provide valuable insights for developing targeted psychological interventions. Moreover, investigating the mediating effects of key variables such as resilience, stress, and recovery in this relationship is paramount. As highlighted by Di Giuseppe et al. [6], resilience could act as a protective buffer against burnout, while stress and recovery dynamics may intricately shape the impact of defense mechanisms. Examining these mediating factors enriches our understanding of the nuanced psychological processes involved, offering a comprehensive perspective that can inform interventions tailored to the specific needs of athletes.

### 1.1. Stress and Sport Burnout

Defined as the body’s response to any demand or challenge, stress in the sporting context arises from various sources, including competitive pressures, performance expectations, and the relentless pursuit of excellence [7]. Athletes frequently encounter stressors related to training intensities, competition outcomes, and the need for consistent peak performance. The transactional model of stress and coping, proposed by Lazarus and Folkman [8], posits that stress is a subjective experience shaped by an individual’s perception and appraisal of environmental demands. Athletes’ cognitive appraisals of stressors, such as the importance and controllability of an event, significantly influence their stress responses [9]. In addition to acute stressors inherent in sports, athletes often grapple with the challenges of chronic stress, a prolonged and sustained state of heightened arousal [7]. Chronic stress can manifest from persistent demands such as rigorous training regimens, continuous performance expectations, and the enduring pressure to meet high standards. The cumulative effect of chronic stress, when not effectively managed, poses a significant risk for athletes, potentially leading to burnout.

In the sports context, burnout can be described as a syndrome characterized by a reduced sense of accomplishment, sport devaluation, and emotional and physical exhaustion [10]. Reduced sense of accomplishment refers to an athlete’s perception of decreased competence and achievement in their sport, often accompanied by a decline in self-esteem [11]. Sport devaluation involves a shift in an athlete’s attitudes and feelings toward their sport, leading to a diminished interest and commitment [5]. Emotional exhaustion, a central component of burnout, manifests as feelings of being emotionally drained and depleted of energy [12]. In the context of Lazarus and Folkman’s transactional model of stress [8], emotional exhaustion can be understood as the result of an individual’s cognitive appraisal process where the emotional demands of a situation are perceived as overwhelming relative to their available emotional resources. Athletes experiencing emotional exhaustion may struggle to cope with the demands of training and competition, ultimately impacting their psychological resilience. Physical exhaustion, on the other hand, refers to the depletion of physical resources, often resulting from intensive training regimens and the pressures of competition [13]. In this perspective, emotional exhaustion is characterized by psychological fatigue that stems from prolonged emotional strain, whereas physical exhaustion results particularly from prolonged physical effort. The detrimental consequences of burnout extend beyond its immediate symptoms, impacting athletes’ overall well-being and performance in the sports arena. Indeed, research in sports psychology has consistently linked burnout to a cascade of adverse outcomes, including compromised physical health, impaired cognitive functioning, and heightened injury susceptibility [14,15]. Recognizing the profound implications of burnout on athletes’ holistic development, there is an increasing emphasis on understanding the determinants of sport burnout to develop targeted preventive strategies [14,15]. In this perspective, exploring the intricate interplay between stress, burnout, and protective factors becomes relevant to foster athlete well-being [16,17].

### 1.2. Recovery and Resilience

Among the protective factors against stress and sport burnout, there could be recovery [18]. Recovery can be defined as an inter- and intra-individual process that occurs over time for the reestablishment of performance abilities [19]. The biopsychological perspective of stress and recovery [20] “embraces physical and biopsychosocial dimensions of both stress and recovery to indicate the extent to which an athlete is physically and/or mentally stressed, as well as whether this athlete is capable of using individual strategies for recovery and which strategies are used” [21]. From there, athletes should focus on keeping and regaining the body’s balance between the imposed demands and the available physical and psychosocial resources, allowing athletes to perform [22]. More particularly, the process of recovery cannot take place solely through the elimination of stress; rather, it includes an action-oriented component (pro-active recovery) designed to optimize the situational conditions (reestablishment of psychological and physical strength) [23]. However, an imbalance between stress and recovery can lead to greater experiences of stress and eventually lead to burnout [22].

To cope against stress, resilience could be considered a highly desirable characteristic for athletes to have in sport [24,25]. Fletcher and Sarkar defined psychological resilience as “the role of mental processes and behavior in promoting personal assets and protecting an individual from the potential negative effect of stressors” [24]. In this theoretical approach, the concept of resilience could be as a dynamic process of bouncing back to normal functioning following stressors [26,27,28,29]. Understanding the bouncing back process requires a focus on the responses of an athlete’s state to stressors over the course of time. This provides insights into, for instance, how quickly athletes return to their normal level following an adverse experience like a heavy defeat. Relatedly, when athletes cannot return to their normal level or the return takes relatively long, it may be a warning signal of a resilience loss [27]. From the Lazarus transactional model [8], resilience represents a key factor that influences how athletes appraise and cope with stress. In the primary appraisal, resilience helps athletes perceive stressful situations as challenges rather than threats. In secondary appraisal, it affects their belief in their ability to cope, fostering more effective coping strategies. This dynamic process of resilience to cope with stress could itself be determined by defense mechanisms.

### 1.3. Defense Mechanisms in Sport

Defense mechanisms are automatic psychological processes that protect an individual from unpleasant emotions and prevent awareness of internal or external danger and stress [30]. Defenses are part of the psychological adaptation process, like coping. However, defense mechanisms and coping strategies have several differences. Indeed, it is generally believed that coping reflects competence-related functioning, whereas defenses are related to internal determinants of functioning [31]. Defenses are considered to be more unconscious and predominantly directed toward inner conflicts, whereas coping is presumed to be relatively conscious and oriented toward outer stressors and adaptation to reality [32,33]. The various defense mechanisms can be organized into a coherent and concise set of dimensions or levels, taking into account their respective levels of maturity. This categorization aims to create a meaningful and parsimonious framework that reflects the developmental sophistication or adaptive nature of each defense mechanism [34,35]. Based on Vaillant’s hierarchical model of defense styles [34], defenses are grouped as mature defenses or adaptive defense mechanisms (ADMs, e.g., altruism or humor) and immature defenses or maladaptive defense mechanisms (MADMs, e.g., projection or acting-out). ADMs enable individuals to effectively process and regulate emotional distress while remaining connected to reality. In contrast, MADMs involve inflexible and exaggerated shifts in personal feelings and/or significant distortions of external reality [36]. Studies focusing on specific defense mechanisms have indicated that certain ADMs, like anticipation and humor, are associated with favorable outcomes, such as enhanced performance. On the contrary, previous research demonstrated that MADMs are associated with detrimental effects [37], such as psychopathology and psychological problems. More particularly, greater reported immature defenses were associated with higher levels of depressive, anxious, personality disorder, and COVID-related post-traumatic stress symptoms [38,39]. For instance, Di Giuseppe et al. [39] showed that dissociation was associated with maladaptive personality types.

In the sport context, several studies have contributed to our understanding of defense mechanisms, shedding light on their intricate interplay with athlete well-being [40,41,42]. In particular, a study showed significant relationships between approach coping and ADM defenses as well as between avoidance coping and MADM defenses [40]. Furthermore, Nicolas et al. [42] showed that ADMs predicted improvement in the use of task coping, pleasant affect, and perceived control and that MADMs predicted the use of competitive avoidance coping, perceived stress, and negative affect. Despite these studies in sport psychology showing the role of defense mechanisms in influencing perceived stress and psychological adaptation to stress, no study to our knowledge has examined the relationship between defense mechanisms and sport burnout. Moreover, it would seem important to study the effects of mediating variables such as stress, recovery, and resilience to better understand the relationship between defense mechanisms and sport burnout. In this perspective, research in the context of health psychology showed significant associations between defense mechanisms, stress, resilience, and burnout [6], but no study to our knowledge has investigated the mediating role of stress, recovery, and resilience in the relationship between defense mechanisms and burnout.

### 1.4. Objectives and Hypotheses

As such, this paper has two objectives: (a) to explore the relationships between ADMs, MADMs, stress, recovery, resilience, and burnout; (b) to examine resilience, stress, and recovery as mediators of the relationship between defense mechanisms and burnout. For the first objective, we expected that (a) ADMs could be negatively linked with stress and burnout and positively linked with resilience and recovery, (b) MADMs could be negatively linked with resilience and recovery and positively linked with stress and burnout, (c) resilience could be negatively linked with stress and burnout and positively linked with recovery, (d) burnout could be negatively linked with recovery and positively linked with stress, and (e) stress could be negatively linked with recovery. For the second objective, we predicted that resilience would mediate the relationship between (a) ADMs and burnout and (b) MADMs and burnout.

## 2. Materials and Methods

### 2.1. Participants

A total of one hundred and seventy-five athletes (68 females and 107 males) ranging in age from 15 to 41 years (*M* = 20.30 years, *SD* = 3.75) participated in this study. Athletes belong to a variety of sports (e.g., athletics, basketball, football, handball, swimming, tennis). On average, they had been practicing in their sport for 9.55 years (*SD* = 5.04) and they trained 6.74 h per week (*SD* = 4.61). They participated in local (*n* = 49), regional (*n* = 73), national (*n* = 46), or international sporting events (*n* = 7).

### 2.2. Measures

The Athlete Burnout Scale (ABO-S) [43] contains fifteen items assessing the three following subscales: physical exhaustion (five items), reduced accomplishment (five items), and negative feelings toward sport (five items). Athletes answered on a 5-point Likert scale ranging from 1 (almost never) to 5 (most of the time). The Cronbach alpha index for this scale was 0.89.

The Short Recovery and Stress Scale (SRSS) is a concise 8-item psychometric tool designed to assess an individual’s perceived stress (4 items) and recovery (4 items) state levels in the sport context. The SRSS represents a short version of the Acute Recovery and Stress Scale for Sport (ARSS) [44] and was developed to satisfy the request for an economic, valid, and change-sensitive psychometric instrument to quantify recovery and stress states. Following the original questionnaire developed by Kellmann et al. [44], the eight items of the ARSS are rated as single items on the same 7-point Likert scale ranging from 0 (does not apply at all) to 6 (fully applies) in relation to their highest ever state. The corresponding adjectives served as descriptors for each item. While the Short Recovery Scale is represented by the items Physical Performance, Capability, Mental Performance Capability, Emotional Balance, and Overall Recovery, and the Short Stress Scale is represented by the items Muscular Stress, Lack of Activation, Negative Emotional State, and Overall Stress. In this study, we used the Overall Recovery (OR) and Stress (OS) dimensions to limit the number of variables in the mediation model. For the recovery scale, Cronbach’s alpha was 0.75, while Cronbach’s alpha was 0.79 for the stress scale.

The Connor–Davidson Resilience Scale-10 (CD-RISC-10) [45] is a shortened version of the original 25-item CD-RISC [46] and designed to assess psychological resilience. The scale consists of 10 items and is structured as a 5-point Likert-type cumulative instrument (0 = never to 4 = almost always). The original version demonstrated a one-dimensional factor structure. A summation of the response to each scale’s item yields a score that ranges from a minimum of 0 to a maximum of 40 that signifies the highest level of resilience. The Cronbach α index of internal consistency was very good, α = 0.85.

The short Defense Style Questionnaire (DSQ-26) represents a tool that allows measuring the defense mechanisms in the sport context [42]. The DSQ-26 comprised 26 items categorized under two subscales: adaptive defense mechanisms (ten items; α = 0.64) and maladaptive defense mechanisms (sixteen items; α = 0.70). Each item was rated on a 6-point Likert scale ranging from 1 (strongly disagree) to 6 (strongly agree).

### 2.3. Procedure

The research was part of the project “Supporting athletes in psychophysiological difficulty: development of interdisciplinary burnout prevention programs” approved by the ethics committee of Alliance Universitaire Bretagne under the number 2303077 and followed the principles of the Declaration of Helsinki. Athletes were recruited face-to-face either directly during a session at two different universities or directly at their training centers, depending on their availability and training location. All individuals practicing a competitive sport during the current sporting season were included. Athletes practicing a sport without an organized competitive cycle (e.g., individual practice in a fitness or strength club) or practicing solely as a non-competitive leisure activity were not included in the study. The same criteria were applied to underage athletes who were approached directly at their training club with the agreement of their coaches and parents. The athletes’ participation was voluntary written informed consent (and parental consent for athletes under 18 years old) was obtained before the data collection. Participants completed the ABO-S, SRSS, CD-RISQ-10, and DSQ-26 on the first half of the competitive season (December 2022). The questionnaires were administered face-to-face only. The Qualtrics survey platform was used to administer the questionnaires to the athletes in the same way. After being briefed on how to answer and complete the questionnaires, the athletes were seated at a table in a quiet room (at the university or in their training center, depending on the location they were recruited). They did not communicate with each other, and an investigator was present throughout the questionnaire administration to answer any questions and ensure technical operation (access to the Qualtrics platform using their smartphone or laptop, with compatibility managed directly by the platform). The questionnaires took around 20 min to administer.

### 2.4. Data Analysis

All the data analyses were performed using the statistical software JAMOVI, version 2.3. We reported the main descriptive statistics (mean, standard deviation). We used skewness and kurtosis indicators with values between −1.96 and +1.96 to establish the normality of the data distribution [47]. Pearson correlation analyses were used to explore the correlation between each normally distributed measure.

Applying a general linear mediation model (i.e., GLM mediation model), we tested the mediation role of OS, OR, and resilience on the relationship between defense mechanisms and burnout. We examined the direct, indirect, and total effects of defense mechanisms, OS, OR, and resilience on burnout. We used the jAMM module, which applies the maximum likelihood estimation method, an optimal procedure for parameter estimations. We calculated the confidence intervals (95% CI) using the large sample delta method, that is, using the z test as the starting point of computation (i.e., the delta method, which extends the approximations from the central limit theorem) [48]. According to the software, a 1000 bootstrap repetition was used.

## 3. Results

### 3.1. Descriptive Statistics and Correlation Analysis

Descriptive statistics and Pearson correlations were obtained to examine the relationships between all continuous study variables (see Table 1). The skewness and kurtosis indicators had acceptable values, ranging between (−1.96 and 1.96) for all variables. Concerning correlation analysis, ADMs were associated positively and significantly with resilience (*r* = 0.545, *p* < 0.001). However, there was no significant correlation between ADM scores and global burnout, stress, and recovery scores. MADMs were associated negatively and significantly with recovery (*r* = −0.198, *p* < 0.01) and negatively and marginally with resilience (*r* = −0.131, *p* < 0.10). MADMs were associated positively and significantly with stress and burnout (*r* = 0.289, *p* < 0.001; *r* = 0.466; *p* < 0.001, respectively). Resilience was associated positively and significantly with OR (*r* = 0.303, *p* < 0.001), whereas resilience was associated negatively and significantly with OS and burnout (*r* = −0.213, *p* < 0.01; *r* = −0.324, *p* < 0.001, respectively). Burnout was associated positively and significantly with OS (*r* = 0.503, *p* < 0.001), whereas burnout was associated negatively and significantly with OR (*r* = −0.448, *p* < 0.001). Finally, OS was associated negatively and significantly with OR (*r* = −0.267, *p* < 0.001).

### 3.2. Mediation Analysis

Direct, indirect, and total effects of the GLM mediation are presented in Table 2. With the introduction of resilience as a mediator, the indirect effect was significant and negative (*β* = −0.129, *p* < 0.01, 95% CI [−0.246, −0.050]), and the components’ regression coefficients were also significant, providing evidence of a mediated effect. Concerning OS and OR as mediators, there were no significant indirect effects. The results showed a significant direct effect of ADMs on burnout (*β* = 0.140, *p* < 0.05, 95% CI [0.010, 0.315]), whereas the total effect of ADMs on burnout was not significant.

Concerning the indirect effects, resilience, OS, and OR were significant mediators of the MADMs and burnout relationship. More precisely, the indirect effects were significant for resilience (*β* = 0.050, *p* < 0.05, 95% CI [0.011, 0.119]), OS (*β* = 0.089, *p* < 0.01, 95% CI [0.044, 0.188]), and OR (*β* = 0.054, *p* < 0.05, 95% CI [0.012, 0.128]). Moreover, the components’ regression coefficients were also significant and showed evidence of a mediated effect. The direct effect of MADMs on burnout was significant (*β* = 0.277, *p* < 0.001, 95% CI [0.209, 0.512]). The total effect of MADMs on burnout was significant (*β* = 0.469, *p* < 0.001, 95% CI [0.438, 0.785]) and higher than the direct effect.

## 4. Discussion

The sporting environment, marked by the sequence of competitions and a relentless pursuit of excellence, exposes athletes to heightened susceptibility to chronic stress, a key precursor to burnout. Exploring the role of defense mechanisms in either exacerbating or mitigating burnout in the sport context could offer valuable insights for crafting tailored interventions. Moreover, examining the mediating effects of key variables such as resilience, stress, and recovery in the link between defense mechanisms and burnout could allow us to better understand this relationship. In this perspective, the aims of the present study were two-fold: (a) to explore the relationships between ADMs, MADMs, OS, OR, resilience, and burnout and (b) to examine resilience, OS, and OR as mediators of the relationship between defense mechanisms and burnout.

### 4.1. First Hypothesis

The results of correlational analyses between ADMs and resilience align with the study by Di Giuseppe et al. [6]. In this context, ADMs such as humor or self-assertion may facilitate overcoming obstacles and adversity, thereby maintaining high performance levels. Interestingly, despite this positive association with resilience, ADM scores did not demonstrate significant correlations with burnout, OS, or OR, suggesting a nuanced relationship that warrants further exploration. From there, it seems particularly interesting to examine the effect of mediating variables such as resilience, stress, and recovery, which could play a role in the ADM and burnout relationship. These findings are also consistent with other research on coping mechanisms that does not support the idea that only one coping strategy is appropriate for each specific situation [41,49]. From this logic, some defenses categorized as adaptive may not be appropriate in certain stressful situations. Moreover, the rigidity of using a defense not appropriate to a specific context could be an additional element in explaining this result. Indeed, research showed that cognitive rigidity plays a role in the development of psychological disorders [50].

MADMs were negatively correlated with OR, indicating that individuals relying on maladaptive mechanisms may experience challenges in achieving effective recovery. Additionally, a marginal negative association was observed between MADMs and resilience, suggesting a potential adverse impact on the ability to cope with adversity. Importantly, MADMs exhibited significant positive correlations with both OS and burnout. These findings are consistent with other research on the detrimental impact of MADMs on psychological distress [6].

Resilience emerged as a crucial factor in the well-being equation, positively and significantly associated with OR. Resilient athletes are often characterized by their proactive orientation toward action [51], showcasing a tendency to engage actively in recovery strategies. Furthermore, research emphasizes the link between resilience and proactive behaviors, confirming the potential positive impact of resilience on athletes’ active involvement in recovery processes [27]. Another interesting finding showed that resilience had negative and significant associations with OS and burnout, reinforcing its role as a protective factor against psychological strain [24]. Indeed, resilient athletes evaluate stressful situations as a motivating challenge, not as a threat [52].

Burnout exhibited a positive and significant association with OS, emphasizing the interconnectedness of these constructs. This reciprocal interaction is consistent with the transactional model of stress and coping, which posits that ongoing stressors can contribute to the emergence and persistence of burnout [8]. The observed negative and significant association between burnout and the OR underscores the intricate interplay between these constructs. Previous research in the field of sports psychology has consistently demonstrated that burnout can compromise athletes’ ability to effectively recover from the physical and psychological demands of training and competition [53]. The reciprocal relationship suggests a vicious cycle, wherein heightened burnout symptoms impede the recuperative process, potentially leading to prolonged fatigue, decreased performance, and increased susceptibility to further stressors [54]. Additionally, OS were found to be negatively and significantly associated with OR, pointing to the potential impact of stress on impeding effective recovery.

### 4.2. Second Hypothesis

The results of the mediation analyses showed a mediation effect of resilience in the relationship between ADMs and burnout. In the analysis of the direct and indirect effects, our findings highlighted that the use of ADMs is associated with an accumulated risk of burnout in the absence of resilience and that, in the presence of resilience, the use of ADMs appears to protect against burnout. These results confirmed that certain defense mechanisms categorized as adaptive may not be appropriate for certain specific situations and therefore their rigid use could cause burnout [41,55]. Moreover, these findings reinforce the idea that the ability to resiliently cope with challenges and stresses can influence how ADMs relate to psychological well-being. From an applied perspective, these results suggest the importance of developing resilience in conjunction with ADMs during burnout prevention interventions.

In the analysis of the indirect effects, results showed a mediation effect of resilience, OS, and OR in the relationship between MADMs and burnout. We also observed a direct effect that was lower than the total effect of MADMs on burnout. The results highlighted that resilience and recovery could attenuate the deleterious effect of MADMs leading to burnout. Indeed, the use of proactive recovery strategies could compensate, to some extent, the influence of problematic mechanisms in stress management [23]. These findings confirmed the importance of taking resilience and recovery into account to prevent burnout. Moreover, the positive indirect effect through OS indicates that MADMs contribute to heightened stress levels, which, in turn, exacerbate burnout symptoms. This is consistent with the transactional model of stress and coping, which posits that maladaptive coping strategies can intensify the experience of stress [8]. The substantial total effect of MADMs on burnout underscores the overall impact of MADMs on athletes’ psychological well-being. This aligns with existing research indicating that reliance on MADM is associated with increased vulnerability to psychological distress and dysfunction [6].

### 4.3. Limits and Perspectives

Notwithstanding the contributions of this research, there were several limitations. First, there is a single measurement time for this study, and it would be interesting to have a longitudinal design in order to assess the chronicity of stress and the stability or not of burnout. For example, it would seem relevant to identify distinct trajectories of athletes’ level of burnout and to examine the influence of defense mechanisms on belonging to these trajectories. Secondly, although our sample of one hundred and seventy-five athletes is adequate for conducting correlation and mediation analyses, it is important to note that larger sample sizes could provide greater statistical power, particularly for detecting smaller effects or more complex interaction patterns. Future studies with larger samples could further confirm the robustness of the current findings and explore additional variables with greater precision. Thirdly, the DSQ used in this study assesses conscious derivatives of defenses. However, one of the most frequent criticisms of self-report questionnaires (quantitative methods) used to assess defenses is that these mechanisms are largely unconscious processes and are thus difficult to assess using a self-report method. In this perspective, future studies should complement self-reported data with an observer-rated method for the assessment of defenses. Fourthly, there were no objective indicators, such as behavioral/biological measures, to evaluate stress and burnout. Since objective indicators are useful to avoid social desirability bias, a future study should test the relationship between defense mechanisms and biological indicators of stress such as heart rate variability or cortisol. Finally, the management of stress represents a multifactorial system with other components that could influence burnout, and it seems relevant to test another mediator, such as cognitive flexibility, in the relationship between defense mechanisms and burnout.

## 5. Conclusions

In conclusion, our investigation has brought new insights to the literature on athlete burnout. Correlation analyses revealed that MADMs were positively associated with burnout, while ADMs had no significant impact on burnout. Our study highlighted that certain defenses categorized as adaptive might not be suitable in specific situations, thus underscoring the influence of mediating variables. In this context, results demonstrated a mediating effect of resilience in the relationship between ADMs and burnout. Consequently, resilience appears to serve as a protective factor against burnout. These findings were further supported by the mediation analyses in the relationship between MADMs and burnout, emphasizing the importance of recovery in burnout prevention. To enhance these practical implications, coaches may consider fostering athletes’ resilience in conjunction with ADMs and implementing targeted psychological exercises to reduce the use of MADMs. Indeed, psychological exercises such as cognitive-behavioral techniques or mindfulness-based practices could help athletes become more aware of these automatic responses [56]. For example, through cognitive restructuring or emotional awareness training, individuals can learn to identify when they are engaging in maladaptive defense mechanisms such as denial or projection and understand how these mechanisms contribute to stress and, eventually, burnout. The goal of these psychological exercises is not to directly modify automatic defense mechanisms, since they are inherently unconscious, but to provide individuals with tools to increase their psychological flexibility. These psychological exercises work by making athletes more aware of their emotional and behavioral responses to stress and helping them develop healthier ways of managing their emotions, thus reducing reliance on maladaptive defenses. However, longitudinal studies and specific interventions are warranted to deepen our understanding of athlete burnout and guide the development of more targeted and effective interventions.

## Figures and Tables

**Table 1:** Descriptive statistics and correlations between study variables.

	Adaptive Defense Mechanisms	Maladaptive Defense Mechanisms	Resilience	Burnout	Overall Stress	Overall Recovery
Mean	4.09	2.69	39.30	2.45	2.39	3.54
SD	0.58	0.51	7.62	0.67	1.41	1.17
Skewness	−0.25	0.34	−0.16	0.52	0.08	0.03
Kurtosis	0.64	−0.06	−0.37	0.07	−0.42	−0.13
Adaptive defense mechanisms	-	-	-	-	-	-
Maladaptive defense mechanisms	0.160 *	-	-	-	-	-
Resilience	0.545 ***	−0.131 ^¥^	-	-	-	-
Burnout	0.056	0.466 ***	−0.324 ***	-	-	-
Stress	0.027	0.289 ***	−0.213 **	0.503 ***	-	-
Recovery	0.059	−0.198 **	0.303 ***	−0.448 ***	−0.267 ***	-

^¥^ *p* < 0.10, * *p* < 0.05, ** *p* < 0.01, *** *p* < 0.001.

**Table 2:** Direct, indirect, and total effects of the GLM mediation.

Type	Effect			95% C.I. ^a^				
		Estimate	SE	Lower	Upper	*β*	*z*	*p*
Indirect	ADMs ⇒ Overal Stress ⇒ Burnout	−0.007	0.026	−0.057	0.043	−0.006	−0.274	0.784
	ADMs ⇒ Overall Recovery ⇒ Burnout	−0.027	0.023	−0.071	0.017	−0.024	−1.196	0.232
	ADMs ⇒ Resilience ⇒ Burnout	−0.148	0.050	−0.246	−0.050	−0.129	−2.954	0.003
	MADMs ⇒ Overall Stress ⇒ Burnout	0.116	0.037	0.044	0.188	0.089	3.147	0.002
	MADMs ⇒ Overall Recovery ⇒ Burnout	0.070	0.030	0.012	0.128	0.054	2.364	0.018
	MADMs ⇒ Resilience ⇒ Burnout	0.065	0.028	0.011	0.119	0.050	2.358	0.018
Component	ADMs ⇒ Overall Stress	−0.049	0.178	−0.397	0.300	−0.020	−0.274	0.784
	Overall Stress ⇒ Burnout	0.144	0.028	0.089	0.199	0.305	5.123	<0.001
	ADMs ⇒ Overall Recovery	0.188	0.151	−0.108	0.484	0.093	1.246	0.213
	Overall Recovery ⇒ Burnout	−0.143	0.034	−0.209	−0.077	−0.253	−4.250	<0.001
	ADMs ⇒ Resilience	7.621	0.813	6.028	9.214	0.581	9.379	<0.001
	Resilience ⇒ Burnout	−0.019	0.006	−0.032	−0.007	−0.223	−3.113	0.002
	MADMs ⇒ Overall Stress	0.807	0.202	0.410	1.204	0.292	3.988	<0.001
	MADMs ⇒ Overall Recovery	−0.488	0.172	−0.825	−0.152	−0.213	−2.844	0.004
	MADMs ⇒ Resilience	−3.340	0.924	−5.152	−1.528	−0.224	−3.613	<0.001
Direct	ADMs ⇒ Burnout	0.161	0.079	0.007	0.315	0.140	2.049	0.041
	MADMs ⇒ Burnout	0.361	0.077	0.209	0.512	0.277	4.657	<0.001
Total	ADMs ⇒ Burnout	−0.021	0.078	−0.174	0.131	−0.018	−0.272	0.786
	MADMs ⇒ Burnout	0.611	0.089	0.438	0.785	0.469	6.907	<0.001

^a^ Confidence intervals computed with the following method: standard (delta method). ADMs = adaptive defense mechanisms. MADMs = maladaptive defense mechanisms.

## Data Availability

The raw data supporting the conclusions of this article will be made available by the authors without undue restrictions.
